# “To Err Is Evolution”: We Need the Implementation Report to Learn

**DOI:** 10.2196/47695

**Published:** 2023-04-04

**Authors:** Caroline Perrin Franck, Antoine Geissbuhler, Christian Lovis

**Affiliations:** 1 Campus Biotech University of Geneva Geneva Switzerland; 2 Geneva Digital Health Hub Geneva Switzerland; 3 Geneva University Hospitals Geneva Switzerland

**Keywords:** implementation science, knowledge management, knowledge sharing, digital health, implementation report

## Abstract

*JMIR Medical Informatics* is pleased to offer *implementation reports* as a new article type. Implementation reports present real-world accounts of the implementation of health technologies and clinical interventions. This new article type is intended to promote the rapid documentation and dissemination of the perspectives and experiences of those involved in implementing digital health interventions and assessing the effectiveness of digital health projects.

## Introduction

The accelerating adoption of digital health, combined with evolving terminology and differing definitions, has created a paradox: there is an exponential increase in knowledge and information, but finding relevant data on implementation processes, and in particular errors or failures, remains a major challenge. This lack of effective documentation of implementation knowledge makes it difficult to reliably study and understand global trends in digital health project failures, exacerbated by a bias toward publishing mostly positive studies [1]. As a result, similar and avoidable mistakes are often repeated.

Digitalization offers numerous opportunities to improve the efficiency and equity of health systems. Yet, many digital health implementations stagnate in the pilot phase or fail to sustain or demonstrate impact. This not only wastes valuable resources but also further fragments already complex health systems, potentially leading to adverse health outcomes [2].

Recurrent errors contribute to inefficient implementation and scale-up, and it is crucial to consider that while new technologies offer immense potential benefits, they can also introduce risks or unintended consequences that have a direct impact on patient outcomes. For example, child mortality increased significantly at one site following the implementation of a commercial computerized physician order entry (CPOE) system [3]. This increase in mortality was primarily due to delayed administration of critical medications [4], amplified by a policy change shortly before implementation, suggesting that policy changes should be avoided during or in close proximity to a CPOE implementation process [5].

However, unintended consequences can also be positive, as demonstrated by the implementation of a telemedicine service in rural Nepal [6]. The service attracted a higher proportion of female patients, possibly due to cultural factors or minimal disruption to their daily lives, which suggests that telemedicine may improve access to health care for female patients [6].

Learning from past implementations is critical. Particularly from an ethical and human rights perspective, as in the absence of established implementation norms and best practices, implementers need to define their own standards for responsible and effective digital health implementations.

## The Potential Transformative Impact of Implementation Reports

Sharing and connecting fragmented knowledge across institutional boundaries can revolutionize an industry. This is evidenced by the aviation sector, a safety-critical industry like health care, where such collaboration has led to transformative results [7]:

Back in the 1930s, flying was really dangerous and passengers were scared away by the many accidents. Flight authorities across the world had understood the potential of commercial passenger air traffic, but they also realized flying had to become safer before most people would dare to try it. In 1944 they all met in Chicago to agree on common rules and signed a contract with a very important Annex 13: a common form for incidents reports, which they agreed to share, so they could all learn from each other’s mistakes. Since then, every crash or incident involving a commercial passenger airplane has been investigated and reported; risk factors have been systematically identified; and improved safety procedures have been adopted worldwide.

The aviation industry has set an exemplary precedent for how sharing mistakes can improve safety and build trust. However, much like the aviation industry in the 1930s, the digital health sector is still in the early stages: the potential to strengthen health care systems is clear, but the bigger picture and potential implications and safety risks are not yet fully understood.

*JMIR Medical Informatics* has created a new article type—the implementation report—to address the challenges associated with managing and effectively sharing implementation knowledge. We need implementation reports to promote greater transparency and accountability; to improve identification of best practices; to optimize resource allocation; and to help gain the trust of patients, practitioners, and other stakeholders. Implementation reports provide a framework for systematically documenting and sharing implementation knowledge, including errors and failures, and present real-world accounts of the implementation of health technologies and clinical interventions. This new article type aims to promote the rapid documentation and dissemination of the perspectives and experiences of those involved in implementing digital health interventions and assessing the effectiveness of digital health projects.

If trial and error is the key engine of evolution, achieving progress requires mechanisms to store and transmit information. DNA is the main information substrate of the evolving living world. We need similar tools, such as the implementation report, to learn and accumulate knowledge from successes and failures in digital health. By embracing the concept that “to err is evolution,” we can collectively harness the power of our experiences to drive innovation and improve health outcomes.

## Abbreviations

CPOE: computerized physician order entry
